# Clinico-pathological significance of extra-nodal spread in special types of breast cancer

**DOI:** 10.7497/j.issn.2095-3941.2014.02.006

**Published:** 2014-06

**Authors:** Ecmel Isik Kaygusuz, Handan Cetiner, Hulya Yavuz

**Affiliations:** Department of Pathology, Zeynep Kamil Maternity and Children’s Training and Research Hospital, Istanbul 34668, Turkey

**Keywords:** Breast cancer, extra-nodal spread, prognosis

## Abstract

**Objective:**

To investigate the significance of extra-nodal spread in special histological sub-types of breast cancer and the relationship of such spread with prognostic parameters.

**Methods:**

A total of 303 breast cancer cases were classified according to tumor type, and each tumor group was subdivided according to age, tumor diameter, lymph node metastasis, extra-nodal spread, vein invasion in the adjacent soft tissue, distant metastasis, and immunohistochemical characteristics [estrogen receptor (ER), progesterone receptor (PR) existence, p53, c-erbB-2, and proliferative rate (Ki-67)]. The 122 cases with extra-nodal spread were clinically followed up.

**Results:**

An extra-nodal spread was observed in 40% (122 cases) of the 303 breast cancer cases. The spread most frequently presented in micro papillary carcinoma histological sub-type (40 cases, 75%), but least frequently presents in mucinous carcinoma (2 cases, 8%). Patients with extra-nodal spread had a high average number of metastatic lymph nodes (8.3) and a high distant metastasis rate (38 cases, 31%) compared with patients without extra-nodal spread.

**Conclusion:**

The existence of extra-nodal spread in the examined breast cancer sub-types has predictive value in forecasting the number of metastatic lymph nodes and the disease prognosis.

## Introduction

Breast carcinoma is the most common malignant tumor and is the leading cause of cancer death in women, with an incidence of more than 90 new cases per pollution of population 100,000 annually. Breast cancer has specific histopathological types with different prognostic and clinical characteristics. Histological type, age, tumor diameter, existing lymph node metastasis, the number of the metastatic lymph nodes, the expression of estrogen receptor (ER) and progesterone receptor (PR), proliferative rate (Ki-67), p53 mutation, and c-erbB-2 are oncogenic-prognostic determinants of breast carcinoma^1^^-^^3^. Both the presence and the total number of tumor-positive lymph nodes are of prognostic value. Grouping patients with one to three, four to nine, and ten or more positive lymph nodes metastases is a generally accepted approach and is used to determine the type of chemotherapy to be given after surgery. Previous studies^4^^-^^8^ assert that in the identification of prognosis, extra-nodal spread has a significant relationship with complete survival, disease-free survival, and local recurrence. This study examines the significance of extra-nodal spread in breast cancer cases and its relationship with prognostic parameters. The significance of the extra-nodal spread in special histological sub-types of breast cancer, as well as its predictive value in guiding treatment, is discussed.

## Materials and methods

A total of 303 cases diagnosed between 1993 and 2004 were selected for the study. The study was approved by the institutional ethics committee. All patients underwent mastectomy and axillary dissection. A total of 100 cases were diagnosed with invasive ductal carcinoma (IDC), 60 with invasive lobular carcinoma (ILC), 37 with pleomorphic lobular carcinoma (PLC), 25 with mucinous carcinoma, 28 with mixed mucinous carcinoma, and 53 with micropapillary carcinoma. After the 303 cases were classified according to tumor type, each tumor group was subdivided according to age, tumor diameter, lymph node metastasis, extra-nodal spread, vascular invasion in the adjacent soft tissue, distant metastasis, and immunohistochemical characteristics.

### Immunohistochemistry

Tumor specimens were fixed in 10% neutral buffered formalin for 20 to 28 h before processing and embedding. Sections were cut on a microtome and stained with H&E. ER, PR, c-erbB-2, and Ki67 were determined through immunohistochemistry by using a streptavidine biotine peroxidase system. Staining protocols for each antibody had previously been optimized and standardized. First, 4 mm thick tissue sections were cut from representative blocks and mounted on charged and pre-cleaned slides. Second, sections were dewaxed in xylene and rehydrated through a decreasing concentration of alcohol to water. Then, heat-induced antigen retrieval (immersion in citrate buffer, pH 6.0, at 95 °C for 30 min followed by cooling at room temperature for 20 min) or, alternatively, a 10 min treatment with protease XIV was applied. Sections were incubated with 0.3% hydrogen peroxide in methanol for 30 min. to block endogenous peroxidase activity. Immunostaining experiments for the localization of ER and PR, p53, c-erbB-2 protein, and Ki-67 antigen were conducted on consecutive tissue sections from the tumor-containing blocks. The primary antibodies used were the monoclonal antibody (Mab) to ER (clone 1D5, at 1/100 dilution; Dako, Glostrup, Denmark), the Mab to PGR (clone 1A6, at 1/800 dilution; Dako), the MIB-1 Mab to the Ki-67 antigen (at 1/1,200 dilution; Immunotech, Marseille, France), and the polyclonal antibody (at 1/3,200 dilution; Dako) to the c-erbB-2 protein.

**Figure 1:**
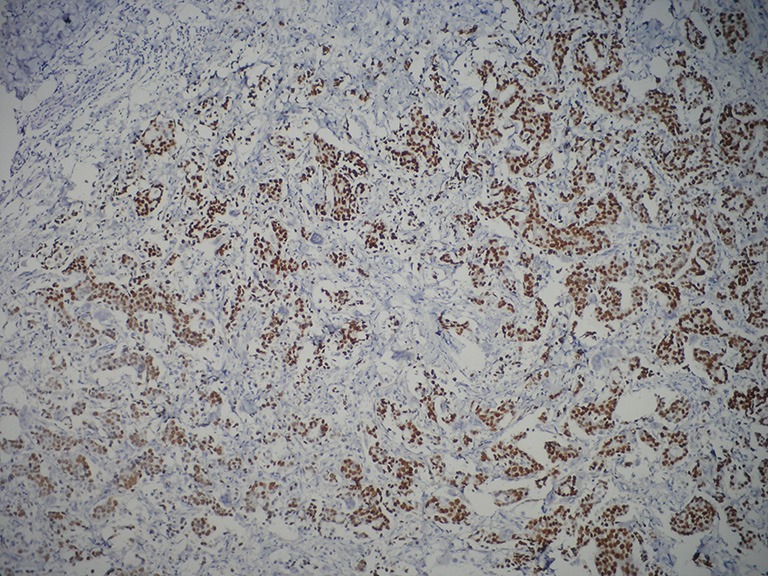
Immunohistochemically ER positive cells (IHC×100). ER, estrogen receptor.

**Figure 2:**
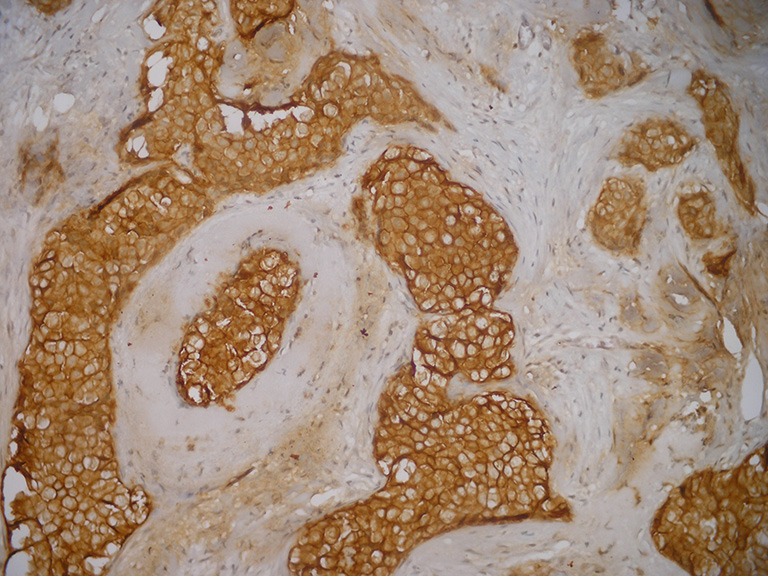
Immunohistochemically c-erbB-2 positive cells (IHC×200).

The immunohistochemically stained slides were evaluated by three pathologists who were all very experienced in breast pathology. Observers were asked to select at least three high-power fields to comprise the spectrum of staining seen on the initial overview. The average percentage of positive stained cells across the section were scored. Only nuclear reactivity, irrespective of its intensity, was considered for ER (**Figure 1**), PR, p53, and Ki-67 antigens, whereas only an intense and complete membrane staining in >10% of the tumor cells qualified for c-erbB-2 overexpression (3+) (**Figure 2**). Steroid hormone receptors status and p53 were classified as negative in case of <1% immunoreactive tumor cells (**Table 1**). The scoring of c-erbB-2 by immunohistochemistry was semiquantitatively evaluated according to the following categories: 0, no membrane staining; 1+, weak and incomplete membrane staining; 2+, weak/moderate heterogeneous complete membrane staining in <10% of tumor cells; or 3+, strong, complete, homogeneous membrane staining in >10% of tumor cells.

**Table 1:** Patients and pathologic charecteristics

Characteristics	Extranodal spread (+)	Extranodal spread (–)	*P*
No. of patients			
Total (303)	122 (40%)	181 (60%)	
IDC (100)	43 (43%)	57 (57%)	
ILC (60)	14 (23%)	46 (77%)	
PLC (37)	14 (38%)	23 (62%)	
Micropapillary (53)	40 (75%)	13 (25%)	
Mucinous (25)	2 (8%)	23 (92%)	
Mixedmucinous (28)	9 (32%)	19 (68%)	
Median age (years)			
Total	58	57	>0.05
IDC	52	54	>0.05
ILC	62	55	>0.05
PLC	60	58	>0.05
Micropapillary	47	52	>0.05
Mucinous	63	67	>0.05
Mixed mucinous	63	59	>0.05
Median tumor size (cm)			
Total	1.9	1.7	>0.05
IDC	2.1	1.7	>0.05
ILC	2.2	1.8	>0.05
PLC	2.2	1.6	<0.05
Micropapillary	2.0	1.6	>0.05
Mucinous	1	1.5	>0.05
Mixed mucinous	1.6	1.7	>0.05
Average number of metastatic lymph node			
Total	8.3	1.2	<0.05
IDC	8.6	0.8	<0.05
ILC	12.3	1.4	<0.05
PLC	11	2	<0.05
Micropapillary	9.7	2.3	<0.05
Mucinous	5.5	0.5	<0.05
Mixed mucinous	2.7	0.4	<0.05
1-3 lymph node metastasis			
Total	33 (11%)	52 (17%)	<0.05
IDC	12 (12%)	15 (15%)	>0.05
ILC	3 (5%)	13 (21%)	<0.05
PLC	6 (16%)	16 (16%)	>0.05
Micropapillary	5 (9%)	7 (13%)	>0.05
Mucinous	1 (5%)	4 (19%)	<0.05
Mixed mucinous	6 (21%)	7 (25%)	>0.05
4-9 lymph node metastasis			
Total	39 (13%)	15 (5%)	<0.05
IDC	17 (17%)	2 (2%)	<0.05
ILC	4 (6%)	3 (5%)	>0.05
PLC	3 (8%)	3 (8%)	>0.05
Micropapillary	11 (20%)	6 (11%)	<0.05
Mucinous	1 (5%)	1 (5%)	>0.05
Mixed mucinous	3 (10%)	0	<0.05
≥10 lymph node metastasis			
Total	39 (13%)	6 (2%)	<0.05
IDC	15 (15%)	1 (1%)	<0.05
ILC	7 (11%)	2 (3%)	<0.05
PLC	7 (19%)	0	<0.05
Micropapillary	9 (17%)	2 (4%)	<0.05
Mucinous	1 (5%)	1 (5%)	>0.05
Mixed mucinous	0	0	>0.05
ER (+)			
Total	49%	41%	>0.05
IDC	38%	37%	>0.05
ILC	52%	38%	>0.05
PLC	56%	46%	>0.05
Micropapillary	45%	45%	>0.05
Mucinous	55%	50%	>0.05
Mixed mucinous	49%	45%	>0.05
PR (+)			
Total	28%	20%	>0.05
IDC	15%	14%	>0.05
ILC	28%	26%	>0.05
PLC	42%	20%	<0.05
Micropapillary	28%	18%	>0.05
Mucinous	35%	21%	>0.05
Mixed mucinous	22%	24%	>0.05
c-erb-B2 (+)			
Total	54%	43%	>0.05
IDC	50%	49%	>0.05
ILC	66%	44%	<0.05
PLC	61%	59%	>0.05
Micropapillary	71%	59%	>0.05
Mucinous	40%	36%	>0.05
Mixed mucinous	54%	43%	>0.05
P53 (+)			
Total	15%	16%	>0.05
IDC	16%	21%	>0.05
ILC	6%	7%	>0.05
PLC	14%	15%	>0.05
Micropapillary	46%	42%	>0.05
Mucinous	8%	3%	<0.05
Mixed mucinous	3%	13%	<0.05
Ki-67 (+)			
Total	19%	16%	>0.05
IDC	25%	23%	>0.05
ILC	18%	15%	>0.05
PLC	23%	20%	>0.05
Micropapillary	24%	19%	>0.05
Mucinous	17%	9%	<0.05
Mixed mucinous	10%	11%	>0.05
Extranodal vascular invasion			
Total	61 (20%)	0	<0.05
IDC	14 (14%)	0	<0.05
ILC	5 (8.5%)	0	<0.05
PLC	2 (7%)	0	<0.05
Micropapillary	23 (45%)	0	<0.05
Mucinous	1 (4%)	0	<0.05
Mixed mucinous	2 (7%)	0	<0.05
Distant metastasis			
Total	38 (31%)	13 (7%)	<0.05
IDC	10 (10%)	5 (3%)	<0.05
ILC	5 (8%)	2 (2%)	<0.05
PLC	5 (13%)	2 (2%)	<0.05
Micropapillary	14 (26%)	3 (5.6%)	<0.05
Mucinous	2 (8%)	1 (2%)	<0.05
Mixed mucinous	2 (7%)	0	<0.05

IDC, invasive ductal carcinoma; ILC, invasive lobular carcinoma; PLC, pleomorphic lobular carcinoma; Ki-67, proliferative rate.

### Clinical follow-up of 122 cases with extra-nodal spread

#### Examined data

**Figure 3:**
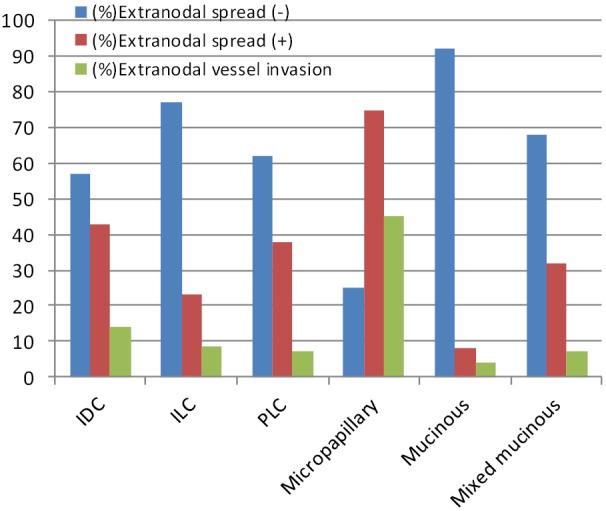
Patients characteristics of with special types breast cancer.
- Prognosis: Patients with extra-nodal spread were reviewed for distant metastases.

- Age: under 50 and above 50;
- Lymph node metastasis: no axillary metastasis, metastasis in one to three lymph nodes, metastasis in four to nine lymph nodes, and metastasis in 10 or more lymph nodes;
- Extra-nodal Spread: Extra capsular growth of tumor cells, invasion of perinodal fat and extra-nodal localization of tumor cells;
- Extra-nodal Vascular Invasion: with and without tumor cells in the vascular section in the extra-nodal adipose tissue (**Figure 3**);

### Statistical analysis

Statistical analysis was performed by using the statistical analysis system. Chi-square and *t*-tests were used. The cases with *P* values under 0.05 were considered significant.

## Results

### Invasive ductal carcinoma (IDC)

The patients with extra-nodal spread had a significantly higher mean number of metastatic lymph node (8.6) and distant metastasis ratio (10 cases, 10%) compared with the patients without extra-nodal spread (*P*<0.05). A significant relationship exists between the extra-nodal spread presence and four or more lymph node metastases (*P*<0.05) (**Figure 3**).

### Invasive lobular carcinoma (ILC)

The patients with extra-nodal spread had significantly higher mean number of metastatic lymph nodes (12.3) and distant metastasis ratio (5 cases, 8.3%) compared with the patients without extra-nodal spread (*P*<0.05). A significant relationship exists between the extra-nodal spread existence and ten or more lymph nodes metastasis (*P*<0.05). C-erbB-2 was highly positive in patients with extra-nodal spread (66%) compared with patients without such spread (44%) (*P*<0.05).

### Pleomorphic lobular carcinoma (PLC)

The patients with extra-nodal spread had a significantly higher mean tumor size (2.2 cm), mean number of metastatic lymph nodes (11), and distant metastasis ratio (5 cases, 13%) compared with the patients without extra-nodal spread (*P*<0.05). A significant relationship exists between extra-nodal spread and the presence of 10 or more lymph nodes metastases (*P*<0.05). PR receptor positivity ratio was significantly high in patients with extra-nodal spread (42%, *P*<0.05).

### Micropapillary carcinoma

Patients with extra-nodal spread had a significantly higher mean number of metastatic lymph node (9.7) and distant metastasis ratio (14 cases, 26%) compared with the patients without extra-nodal spread (*P*<0.05). A significant relationship exists between the extra-nodal spread and the existence of four or more lymph nodes metastasis (*P*<0.05). The number of the lymph node metastasis was higher in the axilla in micropapillary carcinoma compared with the cases diagnosed with mucinous and mixed mucinous carcinoma (*P*<0.05).

### Mucinous carcinoma

Patients with extra-nodal spread had a significantly higher mean number of metastatic lymph node (5.5) and the distant metastasis ratio (2 cases, 8%) compared with the patients without extra-nodal spread (*P*<0.05). In the mucinous carcinoma cases with extra-nodal spread, p53 was significantly more positive than that in cases without extra-nodal spread (*P*<0.05). The cases diagnosed with mucinous carcinoma had the least ratio (8%) of extra-nodal spread among all groups (*P*<0.05). Compared with IDC, mucinous carcinoma was observed in the older population (*P*<0.05).

### Mixed mucinous carcinoma

Patients with extra-nodal spread had a significantly higher mean number of metastatic lymph node (2.7%) and distant metastasis ratio (2 cases, 7%) compared with the patients without extra-nodal spread (*P*<0.05). A significant relationship exists between extra-nodal spread and the existence of 4-9 lymph node metastases (*P*<0.05). C-erbB-2 was highly positive in the mixed mucinous tumors with extra-nodal spread, whereas p53 was interestingly high in patients without extra-nodal spread (*P*<0.05).

### Considering all the cases together as a whole (Table 2)

**Table 2:** Association of the extranodal spread (+) and (-) cases with other prognostic factors

	Extranodal spread (+)	Extranodal spread (–)	*P*
No. of patients	122 (40%)	181 (60%)	
Average age (years)	58	57	>0.05
Average tumour size (cm)	1.9	1.7	>0.05
Average number of metastatic lymph node	8.3	1.2	<0.05
Metastasis in 1-3 lymph nodes	33 (27%)	52 (28.7%)	<0.05
Metastasis in 4-9 lymph nodes	39 (31.9%)	15 (8.2%)	<0.05
Metastasis in 10 and more lymph nodes	39 (31.9%)	6 (3.3%)	<0.05
ER (%)	49	41	>0.05
PR (%)	28	20	>0.05
c-erbB-2 (%)	54	43	>0.05
P53 (%)	15	16	>0.05
Ki-67 (%)	19	16	>0.05
Extranodal vascular invasion	61 (50%)	0	<0.05
Distant metastasis	38 (31%)	13 (7%)	<0.05

ER, estrogen receptor; PR, progesterone receptor; Ki-67, proliferative rate.

Extra-nodal spread was found in 40% (122 cases) of 303 breast cancer cases.

While the extra-nodal spread most frequently presents in the histological sub-type of micropapillary carcinoma (40 cases, 75%), it least frequently presents in mucinous carcinoma (2 cases, 8%).

Patients with extra-nodal spreads had higher mean number of metastatic lymph nodes (8.3) and distant metastasis ratio (28 cases, 9%) compared with the patients without extra-nodal spread.

A relationship exists between the extra-nodal spread and the presence of four or more lymph node metastases (*P*<0.05), as well as between the number of metastatic lymph nodes (≥4) and tumor diameter, extra-nodal spread, distant metastasis, and extra-nodal vascular invasion presence (*P*<0.05). Apart from the tumor type, the presence of distant metastasis was associated with extra-nodal spread.

The relationship between the extra-nodal vascular invasion and the distant metastasis was investigated. However, the results had a limit value (*P*=0.061).

## Discussion

One of the most important prognostic factors in breast cancer is the regional lymph node status^9^. Previous studies^10^^-^^13^ observed that the extra-nodal spread occurred with disseminated axillary lymph node metastases. Vicini *et al*.^13^ proved that patients with extra-nodal spread had significantly more mean metastatic lymph nodes. The mean number of metastatic lymph nodes of the patients with extra-nodal spread in this study was 8.6 in the IDC group, 12.36 in ILC, 11 in PLC, 9.7 in micropapillary carcinoma, 5.5 in mucinous carcinoma, and 2 in mixed mucinous carcinoma. Meanwhile, the mean number of metastatic lymph nodes was 8.3 in patients with extra-nodal spread and 1.2 in the patients without extra-nodal spread.

Patients were divided into three groups according to the number of metastatic lymph nodes (1-3, 4-9, ≥10). A significant relationship was found between extra-nodal spread and the presence of four or more lymph nodes metastases (*P*<0.05) in the IDC, micropapillary, and mixed mucinous carcinoma groups. The ILC, PLC, and mucinous carcinoma groups exhibited a significant relationship between extra-nodal spread and the presence of ten or more lymph node metastases (*P*<0.05). Previous studies^10^^-^^13^ emphasized that the number of metastatic axillary lymph nodes was four or more in patients with extra-nodal spread. The exclusion of extra-nodal invasion by recent TNM versions seems reasonable, mainly because of its direct association with the number of positive lymph nodes and the lack of independent prognostic significance.

Although Leonard *et al*.^11^, Fisher *et al*.^14^, and Mignano *et al*.^4^ stated that the extra-nodal spread affected survival, whereas Donegan *et al*.^10^ and Pierce *et al*.^12^ suggested that it was not. The results of our study show a significant relationship between distant metastases and extra-nodal spread (*P*<0.05). Notably, distant metastases were observed in 31% of the patients with extra-nodal spread but in only 7% of the patients without extra-nodal spread.

A significant relationship between the tumor diameter and the extra-nodal spread was only observed in the PLC group (*P*<0.05).

However, a significant relationship between tumor diameter and the presence of four or more metastatic lymph nodes was observed in all groups, which suggests that extra-nodal spread might have an indirect relationship with the tumor diameter, which is a prognostic factor.

The cases were also related to the presence of vascular invasion on the extra-nodal area in patients. Vascular invasion on adjacent soft tissue was observed in 20% of all patients with extra-nodal spread (61 cases), 14% in IDC (14 cases), 8.5% in ILC (5 cases), 7.1% in PLC (2 cases), 45% in micropapillary carcinoma (23 cases), 4% in mucinous carcinoma (1 case), and 7% in mixed mucinous carcinoma (2 cases) (**Table 2**). The results were significantly when compared with the findings on patients without extra-nodal spread (no extra-nodal vascular invasion was observed) (*P*<0.05). The relationship between extra-nodal vein invasion and distant metastases was also researched, but the results had a limit value (*P*=0.061).

In this study, no axillary recurrence was observed. Previous studies observed axillary recurrence to be 0% (9) or at a very low level of 3% (4 and 10) in cases with extra-nodal spread and no radiotherapy.

Local recurrence was observed in only four (10%) (two IDC, one PLC, one micropapillary carcinoma) of 38 cases that received partial mastectomy. Bucci *et al*.^5^ also failed to detect local recurrence in any of their 43 patients with extra-nodal spread who received adjuvant radiotherapy. Certainly, the low local recurrence ratio in our cases can be attributed to the adjuvant radiotherapy used on all patients. The current findings show that the presence of extra-nodal spread in the examined breast cancer sub-types has predictive value in forecasting the number of metastatic lymph nodes and in disease prognosis.
